# Association between pazopanib exposure and safety in Japanese patients with renal cell carcinoma or soft tissue sarcoma

**DOI:** 10.1038/s41598-023-28688-9

**Published:** 2023-02-06

**Authors:** Takeshi Aoyama, Kenji Nakano, Takeshi Yuasa, Erika Sugiyama, Takako Okawa, Kazuyuki Ito, Keiichi Azuma, Koki Hashimoto, Ryota Furutani, Makoto Hiraide, Kazuo Kobayashi, Kenichi Suzuki, Jyunnichi Tomomatsu, Masataka Tajima, Hitoshi Sato, Toshihiro Hama, Shunji Takahashi

**Affiliations:** 1 Department of Pharmacy, Cancer Institute Hospital, Japanese Foundation for Cancer Research, 3-8-31Ariake, Koto-Ku, Tokyo, 135-8550 Japan; 2 Department of Medical Oncology, The Cancer Institute Hospital, Japanese Foundation for Cancer Research, 3-8-31 Ariake, Koto-Ku, Tokyo, 135-8550 Japan; 3 Department of Urology, The Cancer Institute Hospital, Japanese Foundation for Cancer Research, 3-8-31 Ariake, Koto-Ku, Tokyo, 135-8550 Japan; 4 Division of Pharmacokinetics and Pharmacodynamics, Department of Pharmacology, Toxicology and Therapeutics, School of Pharmacy, Showa University, 1-5-8 Hatanodai, Shinagawa-Ku, Tokyo, 142-8555 Japan; 5 Hoshi University Division of Applied Pharmaceutical Education and Research Ebara, 2-4-41, Shinagawa-Ku, Tokyo, 142–8501 Japan

**Keywords:** Cancer, Oncology

## Abstract

The safety and effectiveness of pazopanib are related to plasma trough concentrations in renal cell carcinoma (RCC); however, data on pazopanib plasma trough concentrations with soft tissue sarcoma (STS) are limited. This study investigated the relationship between plasma trough concentrations and pazopanib safety in 45 Japanese patients with RCC or STS. Among the 33 patients included, the median pazopanib trough concentration was 37.5 (range, 12.1–67.6) µg/mL, which was not significantly different between Japanese RCC and STS patients. The plasma trough concentrations showed significant and positive correlations with aspartate aminotransferase and alanine aminotransferase values in blood samples taken for pharmacokinetic measurements after the administration. The incidence of pazopanib treatment discontinuation were significantly higher in RCC patients (*p* = 0.027). The primary reason for treatment discontinuation was hepatic dysfunction (5/6, 83.3%). Furthermore, this study revealed that pazopanib trough concentration was affected significantly by proton pump inhibitors but not by histamine 2-receptor blockers. In conclusion, the observed pazopanib trough levels and their safety in the Japanese RCC and STS populations in this study were similar to those of the global population. This is the first study to correlate the hepatotoxicity and pharmacokinetic property of pazopanib plasma trough levels by comparing Japanese patients with RCC or STS.

## Introduction

Pazopanib, an angiogenesis inhibitor, is a multi-targeted inhibitor of vascular endothelial growth factor receptors-1,2,3, platelet-derived growth factor receptor α/β, fibroblast growth factor receptor, and stem cell receptor/c-Kit^1^. Pazopanib reportedly improves progression-free survival (PFS) in renal cell carcinoma (RCC) and metastatic soft tissue sarcoma (STS) compared to placebo. Therefore, it has been approved for treating these tumors in Japan^2,3^.

Pazopanib is primarily metabolized by cytochrome P450 3A4 (CYP3A4). Its pharmacokinetics show significant inter-individual variability in plasma exposure^4,5^ and is affected by various factors such as concomitant medication that influences gastric pH and inhibits/induces CYP3A4, food, medication compliance, the timing of drug ingestion, and blood sampling^5–8^. In patients with RCC, a retrospective study showed that the median PFS was significantly prolonged in patients with pazopanib plasma trough concentrations > 20.5 µg/mL than in those with lower concentrations (PFS, 52.0 *vs.* 19.6 weeks, *n* = 177, *p* = 0.004)^9^. In addition, the incidence of adverse effects, such as increased mean arterial blood pressure, diarrhea, hair color change, increase in alanine aminotransferase (ALT) level, stomatitis, and hand-foot syndrome increased based on the pazopanib trough concentrations, such that the highest incidence of adverse effects was observed in the fourth quartile of pazopanib trough concentrations^9^. Thus, in RCC patients, pazopanib has been reported to have a relationship between its blood concentrations and efficacy/safety. However, there has been little information of the pazopanib trough concentrations in STS patients. Some clinical studies also showed that RCC and STS patients have different profiles of adverse events such as increase of liver enzymes^2,3,9^. A previous study did not report an apparent difference in the pharmacokinetics of pazopanib between Japanese and foreign populations^10^. However, this analysis included only a small number of Japanese cases. Furthermore, information on pazopanib or its pharmacokinetics in Japanese patients is limited^11–13^. Hence, it is crucial to investigate pazopanib plasma trough concentration in Japanese patients with RCC and STS.

In this study, we prospectively investigated pazopanib plasma trough concentrations in Japanese patients with RCC and STS. Further, we evaluated the patient factors affecting plasma trough concentrations and the relationship between pazopanib plasma trough concentrations and their safety in these patients.

## Results

### Patient characteristics

**Figure 1:**
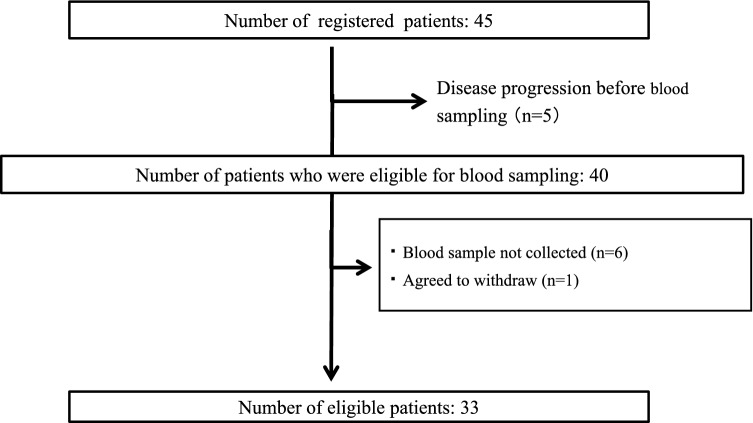
Flow diagram illustrating patient selection.

Of the 45 patients enrolled in this study, 33 were included in the analysis. Of these, 14 had RCC and 19 had STS (Fig. 1). Twelve patients were excluded for the following reasons: disease progressed before blood sampling (n = 5); blood sample not collected (n = 6); and agreed to withdraw (n = 1). Pazopanib was taken in the morning or daytime of the day before the blood sampling day in most patients (27/33, 81.8%), while 6 of 33 patients took it at night.

Table 1 shows a complete overview of patient characteristics at the initiation of pazopanib treatment (baseline), including age, body surface area (BSA), sex, creatinine clearance (Ccr), aspartate aminotransferase (AST), ALT, total bilirubin (T.Bil), and administration of antacids. Ccr, AST, ALT, and T.Bil were in the normal range in both groups. Regarding gender, there were more males in RCC (Male/Female 13/1) compared to STS (8/11) (*p* = 0.004). Regarding the co-administration of antacids, 7 patients were consuming proton pump inhibitor (PPI) (5 used lansoprazole and 2 used vonoprazan fumarate), and 6 were consuming histamine 2 (H2) -receptor blockers (famotidine) for both diseases. One of them had previously undergone gastrectomy.

**Table 1:** Patient characteristics.

	RCC	STS
	n = 14	n = 19
Age (years), median range	67.5 52–74	39 21–75
BSA(m^2^), median range	1.77 1.53–2.32	1.67 1.30–2.20
Sex, Male/Female	13/1	8/11
Ccr (mL/min), median range	79.0 39–189	119 44.2–190
AST (IU/L), median range	19.5 14–43	22 12–60
ALT (IU/L), median range	18.5 9–34	22 7–130
T.Bil (mg/dL), median range	0.6 0.2–0.9	0.40 0.2–1.7
ALB (g/dL), median range	4.2 3.1–4.5	4.2 2.1–48
Administration of antacids		
No antacids	7	13
PPI, yes	3	4
H2-receptor blocker, yes	4	2

BSA, Body surface area; CCr, Creatinine clearance; AST, Aspartate aminotransferase; ALT, Alanine aminotransferase; T.Bil, Total bilirubin; ALB, Albumin; PPI, Proton pump inhibitor; H_2_ blocker, Histamine 2 blocker.

### Pazopanib plasma trough concentrations in patients with RCC or STS

The pazopanib plasma trough concentrations of the 33 patients examined in the study varied individually, with a median of 37.5 μg/mL (range: 12.1–67.6 μg/mL). Most blood samples (29/33, 87.8%) were collected 22 – 40 days (median; 27 days) after the treatment initiation.

**Figure 2:**
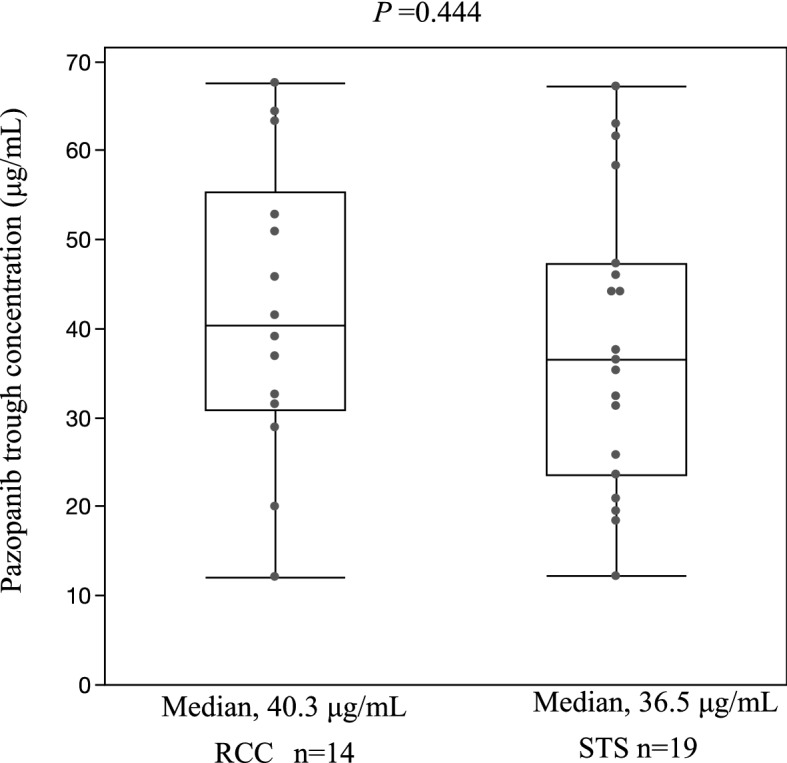
Association of pazopanib plasma trough concentrations with antacid administration in patients with renal cell carcinoma and soft tissue carcinoma. Statistical tests were compared using a Mann–Whitney U test (two-sided).

In RCC patients, the pazopanib median plasma trough concentration was 40.3 μg/mL (range: 12.1–67.6 μg/mL), while it was 36.5 μg/mL (range: 12.1–67.2 μg/mL) in STS patients. Pazopanib plasma trough levels between RCC and STS patients were not significantly different (*p* = 0.444) (Fig. 2). Of the 33 patients, 28 patients (84.8%) had pazopanib plasma trough levels of 20.5 μg/mL or higher [12 (85.7%) and 16 (84.2%) for RCC and STS, respectively].

### Patient baseline background factors and pazopanib plasma trough concentrations

Supplementary Table S1 shows the results of a univariate correlation analysis between patient background factors such as various biochemical test values at the initiation of pazopanib therapy and the acquired plasma trough concentrations of the 33 enrolled patients. The univariate analysis showed no significant correlation between plasma trough levels of pazopanib and the baseline patient background factors. Moreover, no significant difference in the pazopanib trough concentrations was observed between male and female subjects in this study (*p* = 0.575).

### Patient factors during treatment and pazopanib plasma trough concentrations

**Figure 3:**
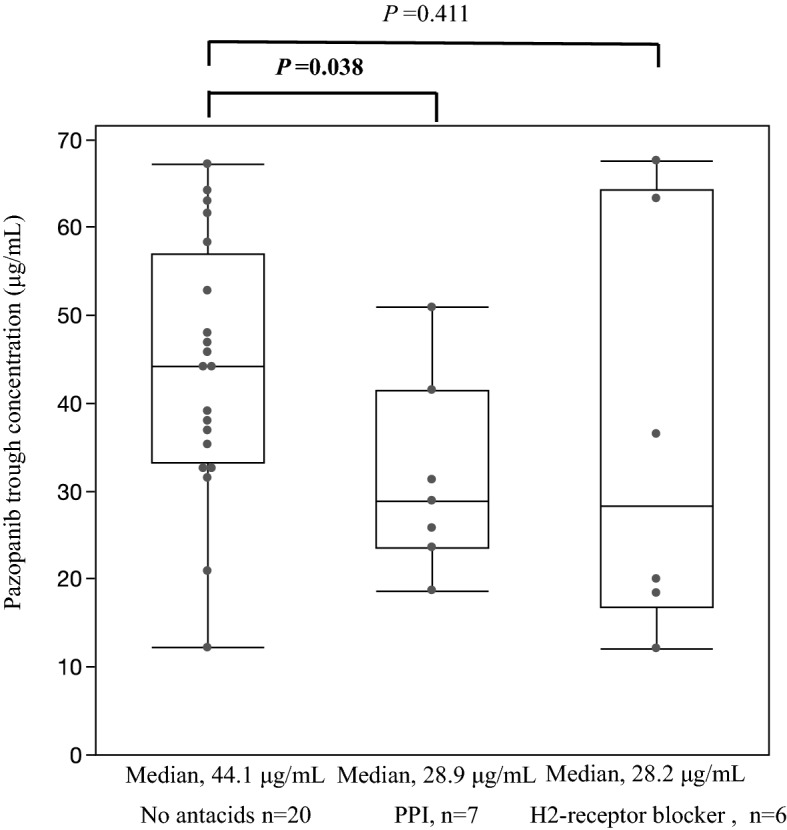
Influence of antacids on pazopanib plasma trough concentrations. PPI, Proton pump inhibitor; H2-receptor blocker, Histamine 2-receptor blocker. Statistical tests were compared using a Mann–Whitney U test (two-sided).

The results of correlation analysis between hepatic function test values at the time of blood sampling for the pharmacokinetic measurement and pazopanib plasma trough concentrations are summarized in Table 2, and Supplementary Fig S1. The univariate analysis revealed that AST and ALT showed significant positive correlations with pazopanib plasma trough concentrations (AST; r = 0.492, *p* = 0.004, ALT; r = 0.434, *p* = 0.012). However, no correlation was observed with T.Bil (r = 0.313). Additionally, pazopanib plasma trough concentrations were significantly lower in patients treated with PPI than in those who did not consume any antacids (*p* = 0.038) (Fig. 3). The median pazopanib plasma trough concentrations of lansoprazole and vonoprazan fumarate were 31.3 μg/mL (range: 18.7–50.9 μg/mL, n = 5), and 26.25 μg/mL (range: 23.6–28.9 μg/mL, n = 2), respectively. Regarding H2-receptor blockers, the plasma trough levels of pazopanib in the six patients taking H2-receptor blockers (including one patient with a history of gastrectomy) were not significantly different from those who did not take antacids (*p* = 0.411). Table 3 shows the adverse event severity by antacid.

**Table 2:** Hepatic function test values during treatment and pazopanib plasma trough concentrations.

	*r*	95% confidence interval	*p*-value
AST (IU/L)	0.492	0.1785–0.7144	0.004
ALT (IU/L)	0.434	0.1071–0.6768	0.012
T.Bil (mg/dL)	0.313	− 0.0339–0.5926	0.076

Statistical tests were compared using univariate correlation analysis*; r* represents Pearson’s correlation coefficient.

AST, Aspartate aminotransferase; ALT, Alanine aminotransferase; T.Bil, Total bilirubin.

**Table 3:** The relationship between antacids and adverse events, treatment discontinuation.

	No antacids (n = 20)	Antacids (n = 13)	*p*-value
Treatment discontinuation, n (%)	4 (20.0)	2 (15.4)	1.000
AST Grade 1/2/3/4	8/3/2/0	7/0/1/0	0.692
ALT Grade 1/2/3/4	7/3/2/0	3/0/1/0	0.341
T.Bil Grade 1/2/3/4	4/2/0/0	0/0/0/0	0.091
Nausea Grade 1/2/3/4	2/0/0/0	4/1/2/0	0.013
Vomiting Grade 1/2/3/4	2/0/0/0	2/1/0/0	0.413
Fatigue Grade 1/2/3/4	8/3/0/0	4/2/0/0	0.889
Hand-foot syndrome Grade 1/2/3/4	6/3/0/0	6/2/0/0	0.712
Diarrhea Grade 1/2/3/4	10/1/1/0	5/1/0/0	0.913
Hypertension Grade 1/2/3/4	1/7/5/0	1/7/3/0	0.537

Two-tailed Fisher’s exact test.

AST, Aspartate aminotransferase; ALT, Alanine aminotransferase; T.Bil, Total bilirubin.

### Adverse events and discontinuation of pazopanib treatment

**Figure 4:**
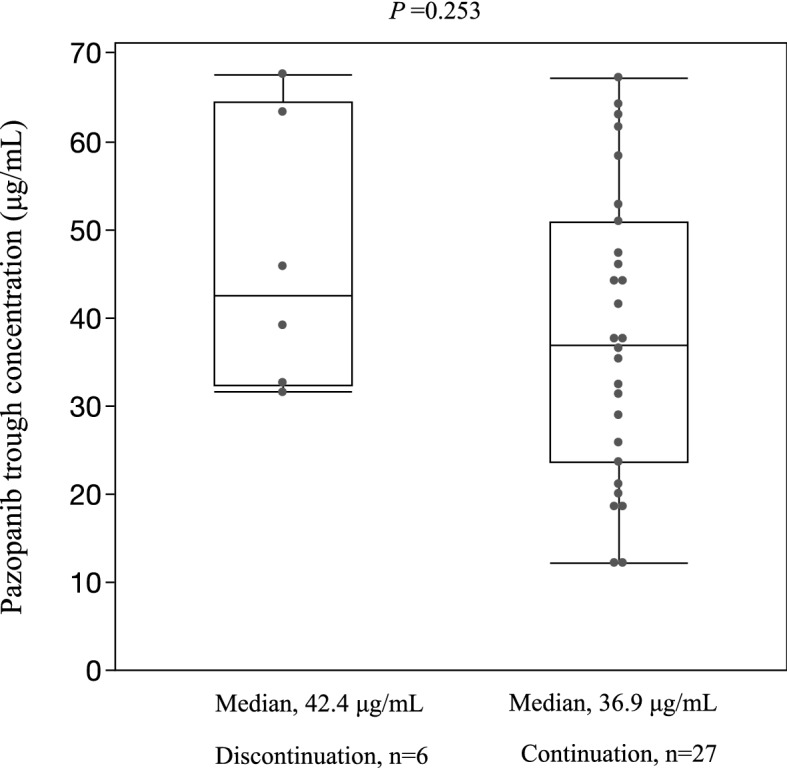
Plasma pazopanib trough levels in patients with discontinued or continued treatment. Statistical tests were compared using a Mann–Whitney U test (two-sided).

Table 4 shows the adverse events leading to treatment discontinuation. Of the 33 included patients, six patients discontinued treatment due to toxicity. The incidence of treatment discontinuation due to adverse events was significantly higher in RCC patients (6/14, 42.9%) than in STS patients (0/19, 0%) (*p* = 0.027). The median duration from treatment initiation to the discontinuation was 40.5 days (range; 25–55 days). The primary reason for discontinuation was hepatotoxicity (5/14, 35.7%), and nausea or vomiting (1/14, 7.1%). Three of the five patients had Grade 3 AST and ALT elevations (Table 4). The median pazopanib plasma trough concentrations in the patients who discontinued the treatment due to toxicity were not significantly different from those who did not (*p* = 0.253) (Fig. 4).

**Table 4:** Treatment discontinuation due to adverse events.

	RCC (n = 14)	STS (n = 19)	*p*-value
Treatment discontinuation, n (%)	6 (42.8)	0 (0)	0.0027
Hepatotoxicity, n (%)	5(35.7)	0 (0)	
AST Grade 1/2/3/4	1/1/3/0		
ALT Grade 1/2/3/4	0/2/3/0		
T.Bil Grade 1/2/3/4	1/1/0/0		
Nausea or vomiting, n (%)	1 (7.1)	0 (0)	
Nausea Grade 1/2/3/4	0/1/0/0		
Vomiting Grade 1/2/3/4	1/0/0/0		

Two-tailed Fisher’s exact test.

RCC, renal cell carcinoma; STS, soft tissue carcinoma.

In addition to hepatotoxicity, some other adverse events including nausea, vomiting, fatigue, hand-foot syndrome, diarrhea, and hypertension were observed during the pazopanib treatment. The pazopanib plasma trough concentrations were compared between the patients with Grade 2 or higher and those with Grade < 2 for these adverse events except vomiting, because only one patient had Grade 2 or higher vomiting. The results showed no significant differences in pazopanib trough concentrations in any of these adverse events (*p* = 0.883, 0.173, 0.190, 0.382, 0.985 for nausea, fatigue, hand-foot syndrome, diarrhea, and hypertension, respectively) (Supplementary Table S2).

Moreover, the relationship between use of antacids and grades of adverse events observed are indicated in Table 3. There were no significant differences in the incidence of the adverse events other than nausea.

## Discussion

This study investigated the pazopanib plasma trough concentrations in Japanese patients with RCC and STS. In both groups, most patients achieved the plasma trough concentrations of pazopanib at 20.5 μg/mL or more, which is considered a threshold indicative of clinical efficacy^9^. We observed significant differences in the plasma trough concentrations of pazopanib (range 12.1–67.6 µg/mL) in individual patients. Also, plasma trough levels did not correlate with patient characteristics such as age, BSA, and biochemical test values at the start of pazopanib therapy. However, they correlated with AST and ALT values acquired on the same day of blood level measurements.The most commonly observed adverse event was elevated hepatic test values (Tables 2 and 4).

The liver is the primary route of the pazopanib metabolic pathway; hence, hepatic dysfunction can be a dose-limiting factor. A phase I study of pazopanib in patients with hepatocellular carcinoma indicated that the most common dose-limiting toxicities were diarrhea, skin hypopigmentation, and AST elevation^15^. In this study, hepatotoxicity was the primary reason for treatment discontinuation (5/6, 83.3%). Treatment-associated elevations in transaminases and bilirubin have also been reported with other tyrosine kinase inhibitors^16^, with the incidence varying with the drug. However, the specific mechanisms involved in elevation remain unknown. This study showed that AST and ALT had significant and positive correlations with pazopanib plasma trough concentrations. However, the correlation coefficient did not indicate a robust correlation (Table 2). The relationship between hepatotoxicity and pazopanib trough levels has been reported; however, ALT levels appeared to reach a plateau at higher concentrations^9^, suggesting not very strong correlations with pazopanib plasma trough concentrations. Additionally, it has also been reported that pazopanib may damage hepatocyte by an immunologic reaction related to genetic mutations in human leukocyte antigen (HLA-B*57:01)^17^, which may also be related to the weak correlation between pazopanib blood levels and AST/ALT. However, in clinical settings, dose reduction of pazopanib is suggested to be effective for AST/ALT elevations^9^, which supports our results. This study did not provide a clear cut-off value of pazopanib plasma trough concentration for liver dysfunction, partly because few patients had Grade 3 or higher hepatotoxicity.

The incidence of hepatotoxicity was significantly higher in RCC patients than in STS patients (Table 4), similar to those of previously reported results^2,3,9^. Additionally, the incidence of treatment discontinuation was higher in RCC patients (Table 4). However, no significant difference was observed in pazopanib plasma trough concentrations between RCC and STS patients (Fig. 2), and it remains unclear why such a difference was observed. Powles et al. reported that ALT elevation induced by pazopanib was associated with older age^18^. In this study, RCC patients were older than STS patients (Table 1), suggesting that this may be one of the reasons for the higher incidence of liver dysfunction in RCC patients. Furthermore, the Ccr level was significantly lower in the RCC group than in the STS group. The possibility that Ccr affected pazopanib plasma trough concentration was examined; however, no correlation was observed (Table S1). The low renal excretion rate of pazopanib (< 4%) may support this finding^19^.

Pazopanib plasma trough concentrations decreased significantly in patients administered lansoprazole and vonoprazan fumarate but not in those administered with an H2-receptor blocker (Fig. 3). Reportedly, pazopanib plasma trough concentration decreases with a PPI, esomeprazole^7^. The dissolution of pazopanib in the gastrointestinal tract decreases under elevated pH conditions due to its physicochemical property, which induces a decrease in its absorption from the gastrointestinal tract. Hence, co-administration with PPIs should be avoided for pazopanib treatment; instead, H2-receptor blockers may be considered for this therapy. Vonoprazan fumarate is not a typical PPI, but a reversible competitive blocker of the H^+^/K^+^ ATPase, an acid pump antagonist. It is known to have strong anti-acidic effect compared to H2-receptor blockers just like conventional PPIs. Since the number of patients receiving vonoprazan fumarate in this study was small (n = 2), we categorized it as the same group with conventional PPIs (lansoprazole). There have been reports of diminished efficacy of pazopanib in patients using PPI or H2 -receptor blockers^20^. However, this previous report did not mention the timing of H2-receptor blocker administration, and pazopanib trough concentrations were not measured. Omeprazole demonstrated an increasing and sustained antacid effect compared to a decreasing antacid effect observed with famotidine^21^. Patients taking H2-receptor blockers may have less effect on pazopanib plasma trough concentration than PPIs. However, 3 of 6 patients taking H2-receptor blockers in this study had blood levels below 20.5 µg/mL. Therefore, the use of H2-receptor blockers may need to be considered if therapeutic benefit is not achieved. Furthermore, in this study, the plasma trough concentration of pazopanib in one patient receiving an H2-receptor blocker and with a history of gastrectomy was as low as 12 µg/mL. Gastric pH may be elevated in some patients with a history of gastrectomy^22^. Hence, attention should be paid to the decrease in blood levels of pazopanib administered with or without PPIs.

Although the antacids can decrease the pazopanib trough concentrations, no significant decrease was observed in incidence of treatment discontinuation and the severity of adverse events in this study (Table 3). Vonoprazan fumarate have been associated with increase in liver enzymes, however, there were no hepatotoxicity discontinuations in the patients receiving vonoprazan fumarate. Regarding nausea, the higher incidence was observed in the patients with antacids. This may be because the antacids were prescribed for nausea to those patients. Thus, it is considered that caution should be taken in adverse events even when antacids are used concomitantly.

This study had a few limitations. First, the study had a small sample size, and we did not find a significant association between pazopanib trough concentrations in those who discontinued or continued treatment. Second, it was challenging to predict pazopanib trough levels associated with the possibility of hepatotoxicity because moderate to severe hepatotoxicity was not observed in any patient. Third, there is a lack of detailed data on the time of intake of the antacids. The timing of oral administrations of antacids may be highly relevant to pazopanib exposure. Fourth, this study showed no associations between pazopanib trough concentrations and the adverse events other than hepatotoxicity. One possible reason for the lack of the associations could be retrospective assessment of the symptoms for some of these adverse events and therefore may be deficient.

In conclusion, once-daily administration of pazopanib 800 mg in patients with RCC and STS, including two patients on a reduced dose of pazopanib (600 mg), resulted in steady-state plasma trough concentrations of approximately 20.5 μg/mL and above. Pazopanib plasma trough concentrations were not significantly different between RCC and STS Japanese patients. However, hepatotoxicity was more common in patients with RCC than in those with STS. Furthermore, this study demonstrated that pazopanib trough concentration was affected significantly by PPI but not by H2- receptors blocker use and that AST and ALT during treatment were correlated with contemporaneous pazopanib trough concentration. To the best of our knowledge, this is the first study to correlate the hepatotoxicity and pharmacokinetic property of pazopanib (i.e., plasma trough level) through comparative investigation among Japanese patients with RCC or STS.

## Methods

### Patient inclusion and data collection

This prospective observational study was conducted at the Cancer Institute Hospital of the Japanese Foundation for Cancer Research. Eligible RCC and STS patients starting pazopanib treatment were recruited after obtaining informed consent regarding their agreement for at least one pazopanib plasma trough concentration measurement. Pazopanib treatment was started at 800 mg/day and administered once daily. Blood samples were collected on or after the 22nd day of pazopanib treatment initiation. When the dose of pazopanib was reduced from the initial 800 mg/day dose, the blood sample was collected at the time of pazopanib dose reduction. Patients who were unable to have their blood collected at the time of pazopanib dose reduction had their blood sampled 14 days or later. The samples included those of two patients receiving a reduced dose of 600 mg/day. The latest pazopanib intake, date, time, dose, and the time of blood sampling were recorded. Patients were excluded from this study if their disease progressed before blood sampling.

Clinical characteristics, including medical history, the reason for pazopanib discontinuation, and adverse events, were collected from their medical records. In order to assess the adverse events during the treatment, biochemical test values such as AST, ALT and T.Bil were measured on the same day as the measurement of pazopanib plasma concentrations. Written informed consent was obtained from all patients. This study was conducted in accordance with the tenets of the World Medical Association Declaration of Helsinki and was reviewed and approved by the Clinical Research Ethics Review Committee of the Cancer Institute Hospital (Approval No. 2014–1091).

### Determination of pazopanib plasma trough concentrations

Plasma trough concentrations of pazopanib were determined using a validated reverse-phase High-Performance Liquid Chromatography (HPLC)–photodiode array method, developed with some improvement of the previous method described by Dziadosz et al.^14^. Briefly, 200 µL of plasma, 30 µL of an internal standard solution (300 µg/mL of canertinib in mobile phase), 20 µL of 1 mol/L HCl, and acetonitrile were vortexed for 5 min and centrifuged at 4 °C, 10,000g for 10 min. The supernatant was filtered using a syringe filter, and 25 µL of the filtrate was injected into the HPLC system. The detection wavelength was set to 265 nm. Acetonitrile and 20 mM phosphate buffer (pH 2.3) were used as the mobile phase in the gradient method at a flow rate of 0.7 mL/min. A good calibration curve for pazopanib plasma concentration was obtained in the range of 10–80 µg/mL (R^2^ > 0.996). The intra- and inter-day variations of this determination method were validated to confirm its reliability.

### Evaluation of pazopanib plasma trough concentrations influencing the patient factors

Univariate analyses were performed to identify the variables influencing pazopanib plasma trough concentrations. On the initiation of pazopanib therapy (baseline values), seven factors, i.e., age, BSA, albumin, AST, ALT, T.Bil, and Ccr, were used for this analysis for each patient. Furthermore, during the pazopanib treatment, hepatic functions, other adverse events and co-administration of antacids, i.e., PPI and H2-receptor blocker, were examined for their correlations with pazopanib plasma trough concentrations.

### Statistical analysis

Statistical analyses were performed using the Mann–Whitney U test for numerical data, and χ2 test or Fisher’s exact test for categorical data, as appropriate. Statistical significance was set at *p-*values below 0.05 (two-sided). The correlation between the two numerical datasets was analyzed using the Pearson correlation analysis for hepatotoxicity. All statistical analyses were performed using JMP^®^ Pro version 15.0.0 (SAS Institute Inc., Cary, NC, USA).

## Supplementary Information

Supplementary Information 1.

Supplementary Information 2.

## Data Availability

The data that support the findings of this study are available from Takeshi Aoyama but restrictions apply to the availability of these data, which were used under license for the current study, and so are not publicly available. Data are however available from the authors upon reasonable request and with permission of Takeshi Aoyama.
