# Supplementary material for: Association between pazopanib exposure and safety in Japanese patients with renal cell carcinoma or soft tissue sarcoma
Source: Sci Rep. 2023 Feb 6;13:2099. doi: 10.1038/s41598-023-28688-9 (PMC9902386; doi:10.1038/s41598-023-28688-9)
Supplement: Supplementary file 1 — Supplementary Information 1. [file 41598_2023_28688_MOESM1_ESM.pdf]

Supplementary Table S1

Patient baseline background factors and pazopanib plasma trough concentrations

	r	95% confidence interval	*p*-value
Age	0.097	-0.254–0.426	0.590
BSA (m^2)	0.141	-0.212–0.462	0.432
Ccr (mL/min)	0.125	-0.227–0.449	0.486
AST (IU/L)	-0.086	-0.417–0.265	0.634
ALT (IU/L)	-0.073	-0.406–0.277	0.685
T.Bil (mg/dL)	0.049	-0.299–0.386	0.785
ALB (g/dL)	0.306	-0.041–0.587	0.083

Statistical tests were compared using univariate correlation analysis; *r* represents Pearson's correlation coefficient.

BSA, Body surface area; CCr, Creatinine clearance; AST, Aspartate aminotransferase; ALT, Alanine aminotransferase; T.Bil, Total bilirubin; ALB, Albumin

Supplementary Table S2

The relationship between pazopanib trough concentration and grade 2≧ of Adverse events

	Median pazopanib trough concentration µg/mL(range) Grade＜2	Median pazopanib trough concentration µg/mL (range) Grade≧2	*p*-value
Nausea	37.2(12.1-67.6)	41.5(20-63.3)	0.883
Vomiting	37.2(12.1-67.6)	63.3(63.3-63.3)	
Fatigue	36.7(12.1-67.6)	47.3(23.6-67.2)	0.173
Hand-foot syndrome	37.2(12.1-63.3)	64.2(20-67.6)	0.190
Diarrhea	37.0(12.1-67.6)	44.0(36.9-63.3)	0.382
Hypertension	44.3(12.1-67.2)	37.2(18.4-67.6)	0.985

Statistical tests were compared using a Mann-Whitney U-test test (two-sided).
