# Supplementary material for: Association between pazopanib exposure and safety in Japanese patients with renal cell carcinoma or soft tissue sarcoma
Source: Sci Rep. 2023 Feb 6;13:2099. doi: 10.1038/s41598-023-28688-9 (PMC9902386; doi:10.1038/s41598-023-28688-9)
Supplement: Supplementary file 2 — Supplementary Information 2. [file 41598_2023_28688_MOESM2_ESM.pdf]

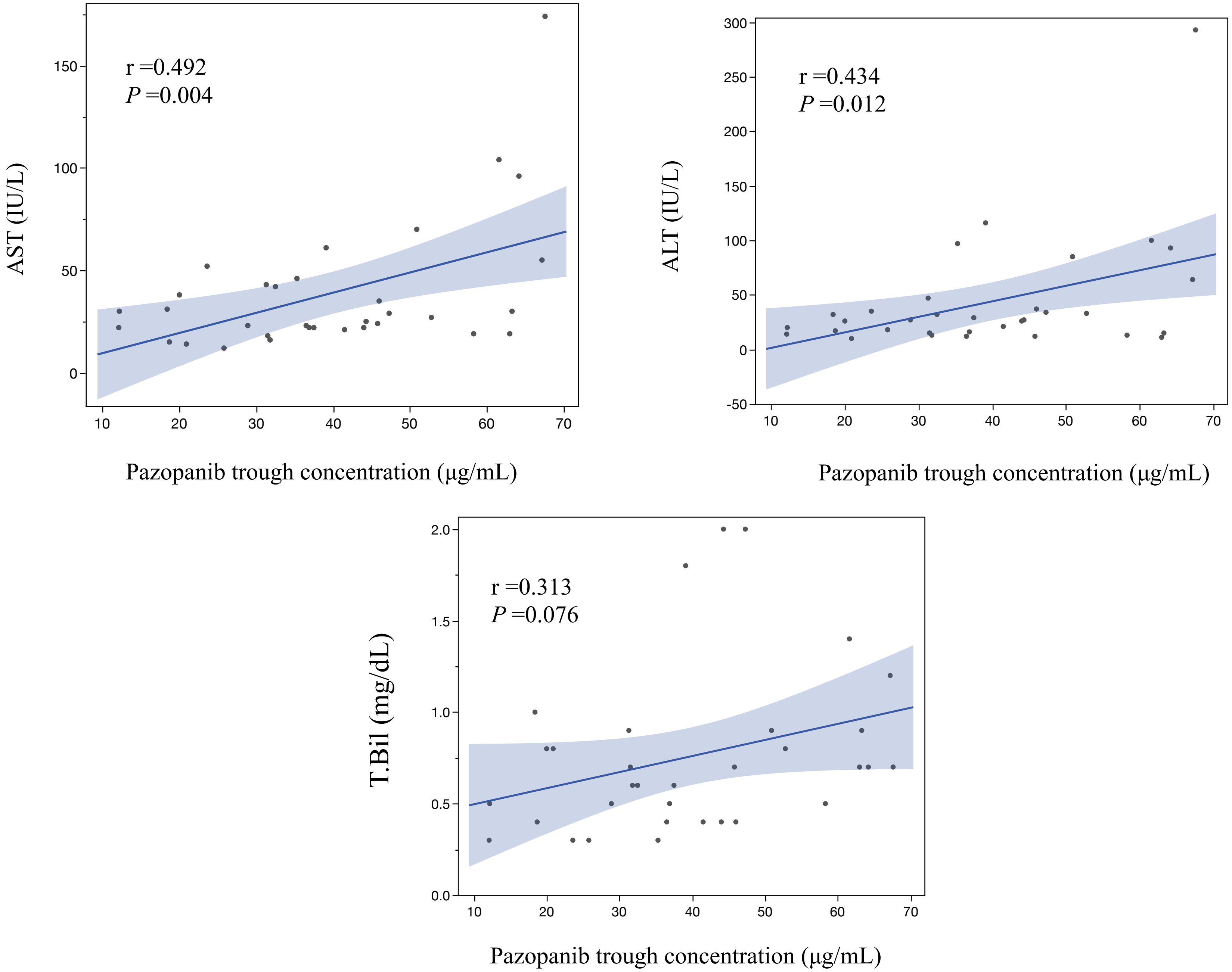

Supplementary Fig S1 Correlation between liver enzyme tests and the pazopanib concentration
